# Supplementary material for: Artesunate Switches Monocytes to an Inflammatory Phenotype with the Ability to Kill Leukemic Cells
Source: Int J Mol Sci. 2021 Jan 9;22(2):608. doi: 10.3390/ijms22020608 (PMC7827848; doi:10.3390/ijms22020608)
Supplement: Supplementary file 1 [file ijms-22-00608-s001.pdf]

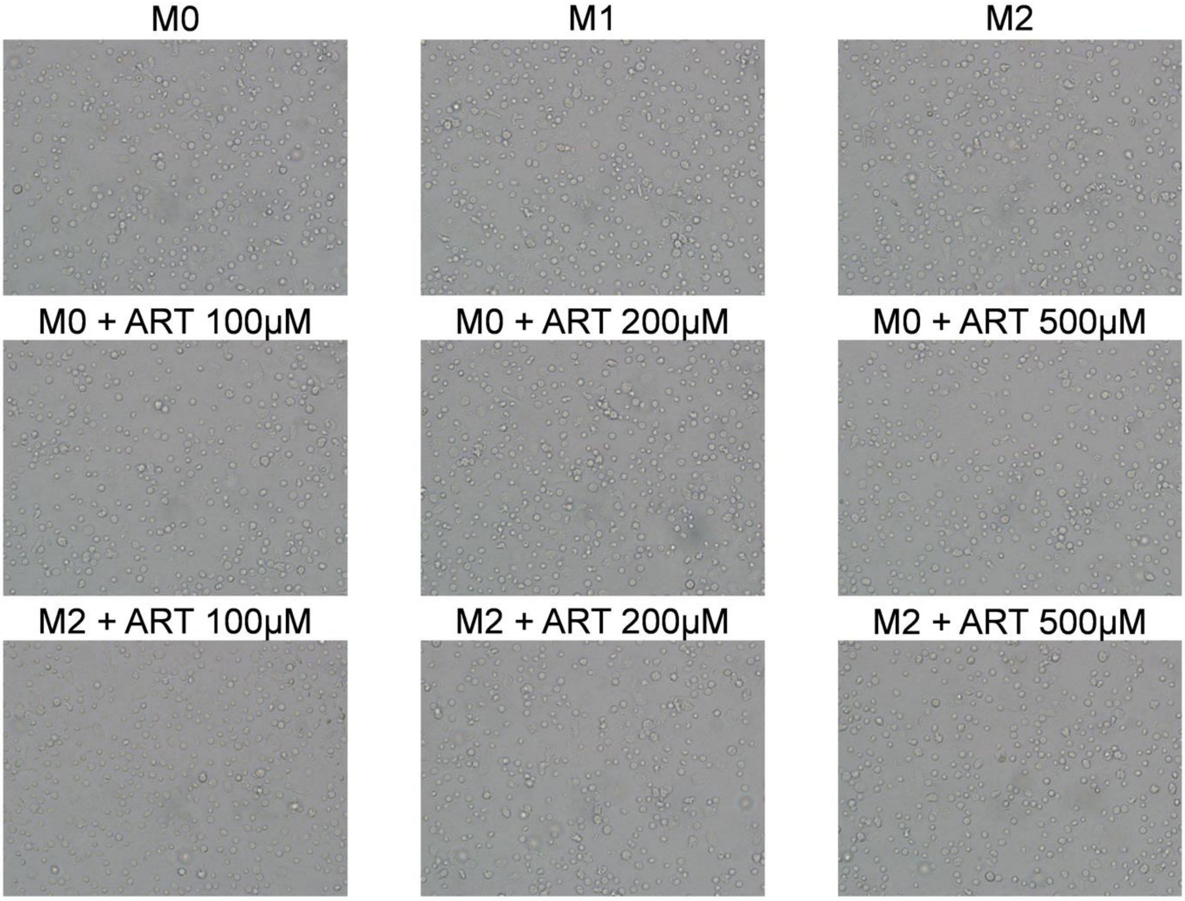

Supplementary Figure S1. Representative light microscopy images of monocytes treated with LPS (10 ng/mL) for M1-type monocytes, IL-4 (20 ng/mL) for M2-type monocytes and both M0 and M2-type monocytes treated with increasing doses of ART.

Supplementary Table S1. List of antibodies and antibody dilutions used in the described experiments.

Antibody	Technique	Reference	Manufacturers	Dilution
CD14-FITC	FC	301804	Biolegend	1:40
CD16-APC	FC	17-0168-42	eBioscience	1:250
CD80-PE	FC	MHCD8004	Invitrogen	1:50
HLA-DR-PerCP	FC	MHLDR31	Invitrogen	1:33
CD163-PerCP	FC	326512	Biolegend	1:33
CD206-APC	FC	321110	Biolegend	1:33
p-JAK2	WB	#3771	Cell Signalling	1:1000
p-STAT3	WB	sc-7993	Santa Cruz	1:1000
STAT3	WB	ab5073	Abcam	1:1000
NF-kB p65	WB	#4764	Cell Signalling	1:1000

p-NF-kB p65	WB	#3033	Cell Signalling	1:1000
CHOP	WB	#2895	Cell Signalling	1:1000
ATF4	WB	#11815	Cell Signalling	1:1000
p-eIF2α	WB	#9721	Cell Signalling	1:1000
IKKα	WB	#2682	Cell Signalling	1:1000
IKKb	WB	#8943	Cell Signalling	1:1000
p-IKKα/β	WB	#2697	Cell Signalling	1:1000
p-JNK	WB	#4668	Cell Signalling	1:1000
p-p38	WB	sc-166182	Santa Cruz	1:1000
p-c-Jun	WB	#2361	Cell Signalling	1:1000
p-ERK	WB	sc-7383	Santa Cruz	1:1000

Legend: WB: Western Blot; FC: Flow Cytometry.
